# Evaluation of different doses of transcutaneous nerve stimulation for pain relief during labour: a randomized controlled trial

**DOI:** 10.1186/s13063-018-3036-2

**Published:** 2018-11-26

**Authors:** Aníbal Báez-Suárez, Estela Martín-Castillo, Josué García-Andújar, José Ángel García-Hernández, María P. Quintana-Montesdeoca, Juan Francisco Loro-Ferrer

**Affiliations:** Department of Medical and Surgical Sciences, University of Las Palmas de Gran Canaria, Paseo Blas Cabrera Felipe, s/n, 35016 Las Palmas de Gran Canaria, Las Palmas, Spain

**Keywords:** Transcutaneous electrical nerve stimulation, Pain relief, Randomized controlled trial, Obstetric labour, Physical therapy modality

## Abstract

**Background:**

Pain during labour is one of the most intense pain that women may experience in their lifetime. There are several non-pharmacological analgesic methods to relieve pain during labour, among them transcutaneous electrical nerve stimulation (TENS). TENS is a low-frequency electrotherapy technique, analgesic type, generally used in musculoskeletal pathology, but it has also come to be used as an alternative treatment during labour. The purpose of this study is to investigate the pain-relieving effect of a TENS application during labour and to find out the most effective dose.

**Methods:**

This study is a randomized, double-blind, placebo-controlled trial. TENS therapy was initiated at the beginning of the active phase of labour. Participants were randomly assigned to three groups (21 per group: two active TENS and one placebo). Active TENS 1 intervention consisted in a constant frequency of 100-Hz, 100-μs, active TENS 2 intervention consisted in a varying high-frequency (80–100 Hz), 350 μs, and in a placebo group, participants were connected to the TENS unit without electrical stimulation. TENS was applied with two self-adhesive electrodes placed parallel to the spinal cord (T10–L1 and S2–S4 levels). The primary outcome was pain intensity (0–10 cm) measured on a visual analogue scale (VAS) at several stages (at baseline and at 10 and 30 min later). Secondary outcomes included women’s satisfaction (via the Care in Obstetrics: Measure for Testing Satisfaction scale).

**Results:**

Sixty-three women participated. Regarding baseline characteristics, no differences were found among the three groups. The active TENS 2 group obtained an improvement with clinically significant VAS results (− 2.9, 95% confidence interval – 4.1 to − 1.6, *p* <  0.001). Regarding satisfaction, the results also revealed better results in the active TENS than in the placebo group.

**Conclusions:**

TENS with high frequencies modified in time as well as high pulse width are effective for relieving labour pain, and they are well considered by pregnant participants.

**Trial registration:**

ClinicalTrials.gov, NCT03137251. Registered on 2 May 2017.

## Background

Pain during labour is one of the most intense types of pain that a woman may experience in her lifetime, and it can be influenced by anatomical and physiological factors and by women’s own experiences, as well as by cultural, social, and environmental factors [1]. Moreover, mothers who experience high levels of pain during pregnancy have an increased risk of complications during labour, like foetal tachycardia, vaginal tears, or alteration in foetal blood samples [2].

Neuraxial analgesia during labour is the most effective method for pain relief, but it appears to be associated with certain side effects, such as maternal hypotension, decreased uteroplacental perfusion, foetal bradycardia, maternal fever and pruritus, an increased oxytocin requirement, a prolonged second stage of labour, a higher rate of caesarean deliveries, and especially, higher costs [3].

Non-pharmacological methods for pain relief include a wide variety of techniques aimed at improving physical sensations and preventing the psychoemotional perception of pain. Among the main non-pharmacological methods of pain relief for childbirth is the application of transcutaneous electrical nerve stimulation (TENS). Its application during childbirth is based on the gait control theory of pain of Melzack and Wall [4]. Furthermore, many non-pharmacological methods of managing pain increase the satisfaction of women with regard to their labour experience [5, 6].

TENS has been used for labour analgesia, and there are several studies which show its effectiveness and safety [7–10]. The effectiveness of TENS depends on the duration, frequency, and amplitude of the stimulating current and the location of the electrodes’ application [11]. Despite the widespread use of TENS and its potential advantages for the relief of labour pain, evidence from systematic reviews has been inconsistent in demonstrating clear benefits of this method, and overall effect for pain relief using TENS in labour was weak [12]. Most of the studies were small or non-randomized trials [13–15]. Furthermore, there is no consensus in the current literature about the exact parameters that allow effective pain relief, and currently there is no common protocol that provides us with an effective clinical practice guide that allows us to be efficient in our intervention.

The aim of this double-blind, randomized, placebo-controlled trial was to investigate the pain-relieving effect of a TENS application during labour and to find out the most effective dose.

## Materials and methods

### Study design

We conducted a randomized, double-blind, placebo-controlled trial. This study (ClinicalTrials.gov ID NCT03137251) was approved by the Hospital’s Human Ethics Committee (ID CEIm-CHUIMI-2016/875), and it followed the ethical guidelines set out in the Declaration of Helsinki. It was also conducted in accordance with the Good Clinical Practice (GCP) guidelines. All patients signed an informed consent statement before starting the study.

When the Hospital’s Human Ethics Committee approved the trial in December 2016, we undertook a pilot study with 20 patients. They did not take part in the final trial and they were excluded for the current analysis. We just wanted to detect possible difficulties in the process; for this reason, these patients were not registered under NCT03137251. Subsequently, 63 participants were enrolled at the Complejo Hospitalario Universitario Insular-Materno Infantil (Spain) between May 2, 2017 and August 30, 2017. The inclusion criteria were as follows: aged above 18, women with a low-risk pregnancy, a gestational age between 37 and 42 weeks, a single foetus, and cervical dilatation of at least 4 cm. Exclusion criteria included the following: aged below 18, a planned caesarean, a high-risk pregnancy, cutaneous damage at the TENS application sites, women wearing a pacemaker or automatic implanted cardiac defibrillator, inability to understand or refusal to sign the informed consent form, and previous experience with TENS.

The sample size and power calculations were performed using the software GRANMO 7.11. Calculations were based on detecting differences of 1.3 units on a 10 numerical pain rate scale at post-data, an alpha level of 0.05, and a desired power of 80%. These assumptions generated a sample size of 63 subjects, 21 per group. Participants in both groups received all other routine obstetric care. The participants were also instructed to choose the most comfortable position. The presence of an accompanying person was permitted during labour and delivery.

Pregnant women who went to childbirth preparation courses were informed about the possibility of using TENS during labour. In this way, most participants had been informed that a clinical trial was being conducted, while the rest of the participants were informed once they were admitted.

Participants were notified about alternative treatments, responsibilities during the study, and the potential advantages and risks associated with this research. Possible side effects caused by this intervention include redness at the electrode sites. However, these symptoms mostly disappear spontaneously within a few days. The people who attended the delivery of the study participants had a minimum of 15 years of experience in the midwifery obstetrics service.

### Randomization and double blinding

Before starting the trial, investigator 1, who was not involved in the selection and inclusion process, assigned a number to each of the three devices designed using different doses (one of them was a placebo). Investigator 2 generated the random sequence (based on simple randomization) by using a computerized random number generator [16]; these processes were concealed from the rest of the staff of the study. At the time of enrolment in the study, each of the 63 participants was randomly assigned to one of three groups, active TENS 1 (*n* = 21), active TENS 2 (*n* = 21), or TENS placebo (*n* = 21). The participants and nurses who evaluated the results were blinded to the group assignments.

To achieve and ensure blinding in the placebo group, participants were connected to the TENS unit in exactly the same way as participants of the active TENS groups. The active indicator of the unit emitted light and sound, but it did not deliver electrical stimulation. In addition to this, the investigator who applied the device did not know if it was the active one or placebo.

### Intervention

**Fig. 1:**
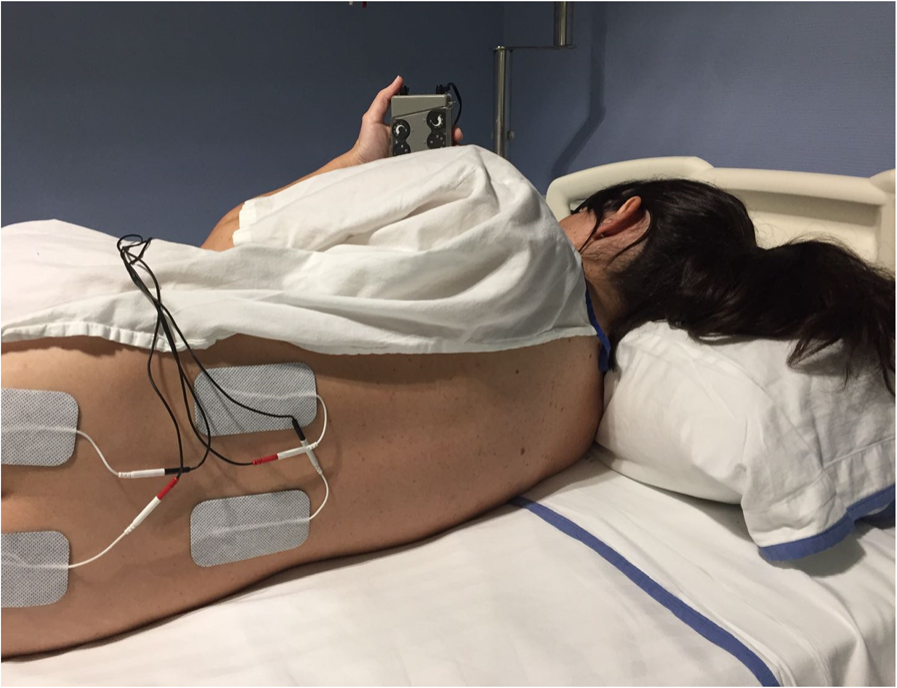
Electrode placement

TENS therapy was initiated at the beginning of the active phase of labour. Investigator 1 programmed the TENS unit and was the only researcher who knew if TENS was active or in placebo mode. The nurses who attended the participants were trained by investigator 1 as study personnel to operate the TENS on the assigned points. However, an external nurse to the obstetrics service entered the data and checked the devices to ensure that the dose administered was always the one programmed in each device. Two pairs of electrodes measuring 5 × 9 cm were fixed on the paravertebral regions of the participants at the T10–L1 and S2–S4 levels (Fig. 1). The TENS device used in this study was a Cefar Rehab 2pro®. In the active TENS 1, it produced a modified biphasic asymmetric pulse, and it was set to a pulse width of 100 μs and a frequency of 100 Hz. In the active TENS 2, it emitted an asymmetric, balanced, biphasic square waveform at a mixed stimulating frequency that randomly varied between 80 and 100 Hz, and it had a pulse duration of 350 μs. The device intensity (amplitude) was individually titrated according to the sensitivity of each parturient. Although this method of determining the level of intensity will result in a variation in delivered amplitude between participants, it is consistent with the techniques of previous literature and clinical practice [17–19]. All groups received TENS constantly over 30 min starting at the beginning of the active phase of labour (4 cm of cervical dilatation). Those women who were comfortable with the TENS were allowed to use it for longer, although the pain relief was only recorded during the first 30 min.

### Primary outcome

The primary outcome was the change in pain severity at the end of the intervention period. The level of pain during labour was measured on a 10-cm-long horizontal linear visual analogical scale (VAS). Baseline VAS evaluations were performed to assess the severity of pain on an intermittent scale from 0 (‘no pain’) to 10 (‘worst pain imaginable’). Evaluations were completed at three distinct stages during the procedure: (1) at the beginning of the active phase of labour, (2) after 10 min, and (3) after 30 min. We considered 1.3 cm the minimal clinically important difference in pain relief [20–23].

None of the participants used analgesic medication during the time from admission to hospital until the end of the revaluation of the pain-related outcomes after the intervention period. This allowed the data from all participants to be included in the analysis of pain outcomes without any possible misleading effects of analgesic medication use.

### Secondary outcomes

The secondary outcome indicators included satisfaction levels and obstetric and neonatal outcomes.

Twenty-four hours postpartum, the second investigator asked participants to answer questions regarding their satisfaction with the care provided. The satisfaction level was measured with the Care in Obstetrics: Measure for Testing Satisfaction (COMFORTS) scale. This scale is a valid and reliable instrument to measure women’s satisfaction with care during labour and the postpartum period [24]. We obtained authorization for using the Spanish version of the COMFORTS scale [25]. It is composed of six subscales: confidence in newborn care, postpartum nursing care, provision of choice, labour and delivery nursing care, physical environment, and respect for privacy. It includes 40 items which participants answered with a 5-point Likert scale in agreement with each statement where 1 = strongly disagree and 5 = strongly agree. The calculation of the results applied to the COMFORTS scale consists of 40 items, and each one of them can be rated from 1 to 5 (1 = strongly disagree and 5 = strongly agree); consequently, the maximum final value is 200 and the minimum value is 40. A level above 171 would be considered a high satisfaction level [24].

### Statistical analysis

Statistical calculations were performed using the IMB SPSS version 18.0 for Windows. The quantitative variables were presented as the mean ± standard deviation. The qualitative variables were presented mediating the absolute frequencies. Statistical methods for analysing differences between groups were one-way analysis of variance (ANOVA) for continuous variables with normal distribution, followed by the χ^2^ test for categorical variables, and a Kruskal-Wallis test when the assumptions of one-way ANOVA were not met. Statistical significance was defined as *p* <  0.05. An external nurse to the obstetrics service entered the data and checked the devices to ensure that the dose administered was always the one programmed in each device. The data was analysed by a statistician who did not intervene in the clinical trial.

## Results

**Fig. 2:**
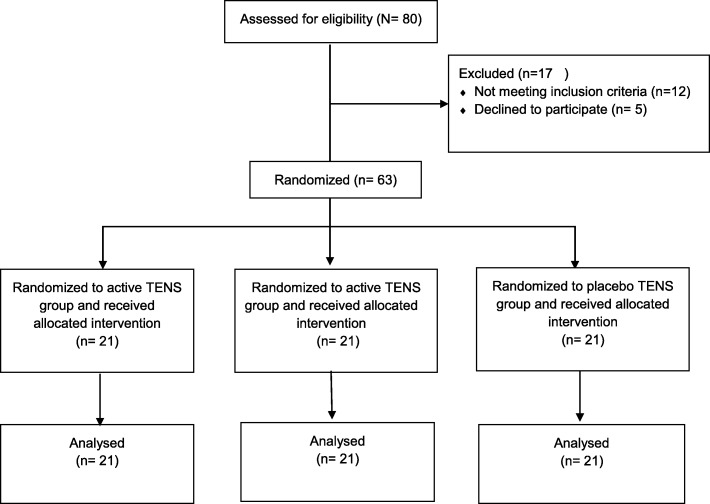
Consolidated Standards of Reporting Trials (CONSORT) flow diagram describing participant allocation in this study

No differences were found among the three groups regarding maternal age, weight, body mass index, gestational age, presentation, childbirth preparation course, position adopted during labour, and kind of pushing during the third stage of labour (Table 1). Figure 2 shows the progression of the participants throughout the trial. There were no dropouts during the study. The baseline characteristics of the participants in each group are presented in Table 1.

**Table 1:** Baseline participant characteristics and obstetric outcomes

Characteristic	Group			*p*
	TENS 1 (*n* = 21)	TENS 2 (*n* = 21)	Placebo (*n* = 21)	
Age (years)	28.3 ± 5.3	28.9 ± 6.0	27.1 ± 5.3	0.545*
Weight (kg)	72.9 ± 10.9	75.9 ± 12.6	71.5 ± 7.7	0.399*
BMI (kg/m^2^)	26.7 ± 2.9	28.7 ± 5.4	26.8 ± 1.6	0.745*
Presentation				0.714**
Cephalic-vertex	12 (19)	11 (17.5)	8 (12.7)	
Cephalic-sinciput	5 (7.9)	3 (4.8)	5 (7.9)	
Cephalic-brow	1 (1.6)	3 (4.8)	4 (6.3)	
Cephalic-face	0	0	1 (1.6)	
Breech	3 (4.8)	4 (6.3)	3 (4.8)	
Gestational age (weeks)	39.5 ± 1.5	39.6 ± 1.5	39.3 ± 1.3	0.508*
Childbirth preparation course				0.446**
Yes	13 (20.6)	11 (17.5)	15 (23.8)	
No	8 (12.7)	10 (15.9)	6 (9.5)	
Length of first stage (min)	443 (216)	451 (238)	527 (225)	0.532*
Length of second stage (min)	42 (23.5)	40 (18.3)	38 (21.4)	0.722*
Position adopted during labour				0.319**
Sitting	3 (4.8)	3 (4.8)	1 (1.6)	
Lateral decubitus	1 (1.6)	2 (3.2)	0	
Dorsal decubitus	7 (11.1)	10 (15.9)	4 (6.3)	
Dorsal decubitus and sitting	6 (9.5)	4 (6.3)	9 (14.3)	
Lateral decubitus and sitting	2 (3.2)	0	3 (4.8)	
Lateral and dorsal decubitus	2 (3.2)	2 (3.2)	4 (6.3)	
Pushing methods for the second stage of labour				0.774**
Valsalva pushing	12 (19)	10 (15.9)	12 (19)	
Spontaneous pushing	9 (14.3)	11 (17.5)	9 (14.3)	
Perineal laceration				0.469**
None	1 (1.6)	1 (1.6)	0	
Grade I	15 (23.8)	19 (30.2)	19 (30.2)	
Grade II	4 (6.3)	1 (1.6)	2 (3.2)	
Grade III	1 (1.6)	0	0	
Accompanying person during the active phase of labour	21 (33.3)	21 (33.3)	21 (33.3)	1**

Data are presented as mean ± standard deviation or *n* (%)

*TENS* transcutaneous electrical nerve stimulation, *BMI* body mass index

*p values obtained from Kruskal-Wallis test and one-way analysis of variance tests

**p values obtained from χ^2^ test

A significant association of VAS was detected depending on the type of TENS over time. The initial pain level of the active TENS 1 group had a mean of 7.4 ± 1.5, the active TENS 2 group a mean of 8.1 ± 1.2, while the TENS placebo group presented a mean of 6.6 ± 1.7 (*p* <  0.05). The women of the TENS 2 group started with a higher level of pain, followed by the TENS 1 group, and the TENS placebo group. The mean VAS pain scores in all the groups at different stages are shown in Table 2.

**Table 2:** Pain visual analogue scale scores at different stages

	Group		
Stage	TENS 1	TENS 2	TENS placebo
Baseline	7.0 ± 1.5	8.1 ± 1.2	6.6 ± 1.7
10 min	6.2 ± 1.4	6.2 ± 2.0	8.3 ± 1.2
30 min	6.3 ± 1.7	5.9 ± 1.9	8.8 ± 1.1
		Difference (95% CI)	p*
Baseline			
TENS 1-TENS 2		−1.0 (− 2.2 to 0.1)	0.079
TENS 1-Placebo		0.4 (− 0.7 to 1.6)	0.668
TENS 2-Placebo		1.5 (0.3 to 2.7)	0.009
10 min			
TENS 1-TENS 2		−0.4 (− 1.3 to 1.2)	0.995
TENS 1-Placebo		−2.1 (− 3.3 to – 0.8)	< 0.001
TENS 2-Placebo		− 2.1 (− 3.3 to – 0.8)	< 0.001
30 min			
TENS 1-TENS 2		0.4 (− 0.7 to 1.7)	0.646
TENS 1-Placebo		− 2.4 (− 3.7 to − 1.5)	< 0.001
TENS 2-Placebo		−2.9 (− 4.1 to − 1.6)	< 0. 001

Data are visual analogue scale mean ± standard deviation scores unless otherwise specified

*TENS* transcutaneous electrical nerve stimulation, *CI* confidence interval

*p values obtained from one-way analysis of variance

Therefore, to correct the possible effect that could be generated when comparing pain at baseline with pain at the end of the intervention, the analysis of covariance (ANCOVA) method was used, detecting a significant association between baseline and after 30 min (*p* < 0.001) as well as with the type of treatment (*p* < 0.001). The global average considering the data of the three groups was 7.269 (Table 3).

**Table 3:** Adjustment of baseline pain level differences using the ANCOVA method

Group	Mean	error*	Difference (95% CI)
TENS 1	6.514^a^	0.300	5.913 to 7.115
TENS 2	5.382^a^	0.315	4.750 to 6.013
Placebo	9.200^a^	0.308	8.583 to 9.816

Data are visual analogue scale (VAS) mean values

*TENS* transcutaneous electrical nerve stimulation, *CI* confidence interval

*error values obtained from ANCOVA method

^a^The covariates that appear in the model are evaluated with the following values: pain VAS at baseline = 7269. The dependent variable is VAS after 30 min

The between-group analysis highlighted a significant decrease in pain, as measured on the VAS, at several stages (baseline, 10 min, and 30 min later) in the active TENS 2 group compared with the TENS 1 group and also compared with the TENS placebo group**.** The only group that obtained an improvement with clinically significant results (more than 1.3 cm of the VAS) was the active TENS 2 group. Therefore, better results were obtained using high frequencies modified in time (80–100 Hz), as well as a high pulse width (350 μs). However, a repeated measures test was analysed during the baseline and at 10 and 30 min after intervention (Table 4).

**Table 4:** Repeated measures test for three measurements (baseline, 10 min, and 30 min)

Group	(A) Time	(B) Time	Differences (A – B)	p*	Difference (95% CI)
TENS 1	1^a^	2^b^	0.810	0.029	0.063 to 1.556
		3^c^	0.667	0.135	− 0.136 to 1.469
	2	1	−0.810	0.029	−1.556 to −0.063
		2	−0.143	1.000	−0.637 to 0.351
	3	1	−0.667	0.135	−1.469 to 0.136
		2	0.143	1.000	−0.351 to 0.637
TENS 2	1	2	−1.857	> 0.001	1.111 to 2.603
		3	2.238	> 0.001	1.436 to 3.040
	2	1	−1.857	> 0.001	−2.603 to − 1.111
		2	0.381	0.187	−0.113 to 0.875
	3	1	−2.238	> 0.001	−3.040 to −1.436
		2	−0.381	0.187	−0.875 to 0.113
Placebo	1	2	−1.762	> 0.001	−2.508 to − 1.016
		3	−2.190	> 0.001	−2.993 to −1.388
	2	1	1.762	> 0.001	1.016 to 2.508
		2	−0.429	0.110	−0.922 to 0.065
	3	1	2.190	> 0.001	1.388 to 2.993
		2	0.429	0.110	−0.065 to 0.922

Data are visual analogue scale mean

*TENS* transcutaneous electrical nerve stimulation, *CI* confidence interval

^*^p values obtained from repeated measures test

^a^Baseline

^b^After 10 min

^c^After 30 min

All anthropometric measures of the newborns are presented in Table 5. The mean values of newborn weight and head circumference were not significantly different between groups. No differences were observed in pain relief with regard to the newborn or mother anthropometric or general characteristics. In all groups, all of the newborns had Apgar scores > 7 by the first minute after birth, and all had normal scores by the fifth minute after birth.

**Table 5:** Anthropometric measures of the newborns

Characteristics of the newborn	Group			p*
	TENS 1 (*n* = 21)	TENS 2 (*n* = 21)	TENS placebo (*n* = 21)	
Weight (g)	3303.0 ± 367.7	3176.1 ± 366.2	3172.8 ± 453.1	0.485
Height (cm)	47.9 ± 1.8	47.5 ± 1.7	48.6 ± 1.6	0.127
Cranial perimeter (cm)	34.7 ± 1.7	33.8 ± 2.0	33.9 ± 1.6	0.254

Data are mean ± standard deviation or *n* unless otherwise specified

*TENS* transcutaneous electrical nerve stimulation

*p values obtained from Kruskal-Wallis test

**Fig. 3:**
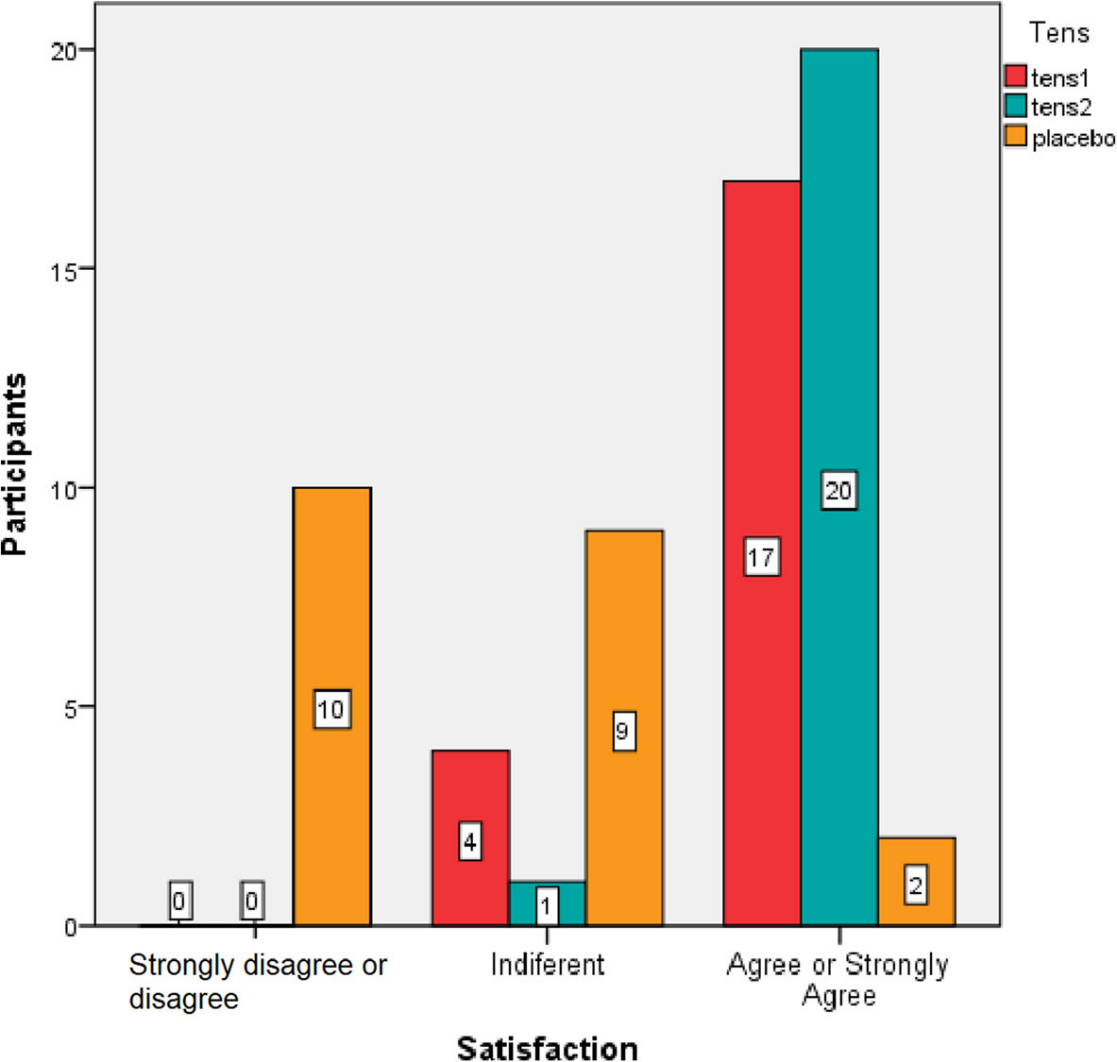
COMFORTS scale. Question 6: Measures to control pain during labour. *TENS* transcutaneous nerve stimulation

To determine the overall satisfaction with the program, we used the COMFORTS scale. The one-way ANOVA test results revealed differences among groups with higher levels of satisfaction in the active TENS groups (active TENS 1, 175.1 ± 11.7; active TENS 2, 177.6 ± 11.3) compared with the TENS placebo group (165.1 ± 9.2). No significant differences between the active TENS groups were observed regarding the labour experience and satisfaction with the care provided during labour. Regarding question 6, which refers to measures to control pain during labour, participants showed a greater degree of satisfaction in the active TENS groups versus the TENS placebo group (Fig. 3).

No patients in any group reported adverse events such as skin allergy or burning at the electrode site.

With regard to the effectiveness of the blinding of the participants and the nurses, responses in the placebo group were not significantly different from those of the active TENS groups (*p* >  0.05), suggesting an adequate blinding in all cases.

## Discussion

The main purpose of this study was to evaluate the pain-relieving effect of TENS during labour and to establish the most effective dose. VAS scores highlighted a decrease in pain in the active TENS groups compared with the placebo group. Moreover, the reduction in pain reached the minimum clinically relevant difference. Regarding satisfaction, results also revealed better results in the active TENS groups than in the placebo group. No adverse effects on the mothers or newborns were recorded.

The findings of the present study in relation to our main objective are similar to those of previous studies. Bundsen et al. [26], Van der Spank et al. [7], and Chao et al. [8] reported a significant reduction in pain intensity in the TENS active group. However, the methods of those studies are very different from those of the present study. Bundsen et al. [26] used the TENS device interchangeably, placed on the lower back, on acupuncture points, and on other parts of the body. Van der Spank et al. [7] used different parameters during the TENS application: a fixed internal frequency of 80 Hz and a burst frequency of 2 Hz, with a pulse duration of 275 μs, thus obtaining a reduction of 1.5 points on the VAS (lower than our results). Chao et al. [8] also used different parameters and applied it to specific acupuncture points.

Our results in terms of quantification of pain reduction showed that there was a decrease in the patients’ pain scores in the active TENS groups compared with the TENS placebo group during the procedure, and it was clinically relevant in the active TENS 2 group. Moreover, the reduction in pain reached the minimum clinically relevant difference (1.3 points on the VAS), as was previously validated for Bernstein et al. [20], Gallagher et al. [21], Todd et al. [22], and Santana et al. [23], who applied the same doses and localization TENS, obtaining an improvement that was almost double that of our study. A possible explanation for these findings may relate to individual pain perception, which in labour depends on the intensity and duration of the contractions, the physical condition of the woman, as well as a complexity of emotional factors, such as previous experiences, present expectations, and cultural factors [27]. No data on these characteristics were collected in our study.

With regard to the TENS location, there is not a definitive consensus to it being applied on the back (Bundsen et al. [26], Van der Spank et al. [7], Santana et al. [23]) or on acupuncture points (Bundsen et al. [26] and Chao et al. [8]). The optimization of TENS depends on accurately selecting the electrode position, current waveform, waveform duration, frequency, and intensity. Prior reports indicate that the greatest degree of pain reduction occurs when the electrodes are placed within the receptive field for the nerve roots to alter nociceptive transmission in the dorsal horn of the spinal cord. In our study, the electrodes were placed parallel to the spinal cord at the T10–L1 and S2–S4 levels (instead of placing them on acupuncture points) to stimulate the nerve roots at the dermatomal level, corresponding to the whole uterus. Saxena et al. [28] compared the efficacy of TENS administered by dermatomal stimulation with TENS administered by stimulation of acupuncture. In their study, TENS administration by dermatomal point stimulation provided early onset and better pain relief in labour. However, it is important to note that authors who applied TENS at acupuncture points recognize that the physiological mechanisms whereby TENS can relieve pain are uncertain.

In our study, satisfaction was significantly higher in the TENS active groups because this intervention resulted in a statistically significant and clinically meaningful reduction in pain. Even in studies where there are not significant differences in pain relief, many of their participants have stated that they would prefer to use TENS for a future labour. A systematic review by Dowswell et al. [10] included 17 randomized, controlled trials comparing women receiving TENS during labour versus routine care or placebo devices. The authors demonstrated little difference in satisfaction with pain relief or in pain ratings between the TENS and control groups. Therefore, it seems reasonable to assume that the use of TENS may contribute to greater acceptance and more frequent use during delivery. In addition, the possibility of including it within the process of routine care should be considered. Despite the results obtained, we must bear in mind that it is a subjective result susceptible to recall bias.

Another factor to take into account with TENS is the accommodation factor. Patients of our study were instructed to increase the TENS intensity to the maximum non-painful level and to report if they perceived any decrease in their stimulus perception (which happens as a result of nerve accommodation). We used high-frequency TENS that randomly varied between 80 and 100 Hz. We based this choice on evidence suggesting that delivering random frequencies provides superior pain relief compared with a conventional fixed frequency [29]. It is considered that applying a stimulus with modulated or alternating frequency reduces the accommodation suffered by the nervous system against monotonous impulses, since with the variation of frequencies the stimulus that the patient is receiving varies continuously [30–32]. Santana et al. [23] used a constant frequency of 100 Hz, observing a significant improvement in pain relief with an application time of 30 min. However, it was observed that if there was a significant improvement in the degree of pain at 15 min, but at 30 min it began to rise, it was probably the result of the accommodation effect.

With regard to the TENS pulse width, Santana et al. [23] applied 100 μs. In our study, we selected 350 microseconds because it has been observed that the increase of the duration of the pulse in 250 microseconds can produce more analgesic effects [33].

The use of TENS during labour has advantages and disadvantages. Advantages of TENS includes non-invasiveness, easy application, no interference with maternal consciousness or mobility, safety, and freedom from any significant side effects [7, 34, 35]. However, there are some indirect side effects that result from the use of neuraxial anaesthesia that can be underestimated. These may include, for example, longer first and second stages of labour, an increased incidence of foetal malposition, and increased use of oxytocin and instrumental vaginal deliveries. In this context, there are not sufficient studies that describe the relationship between TENS and lacerations. Tischendorf et al. [36] describes an incidence of episiotomy or lacerations of 52%, and we found in our study an 87% incidence of superficial vaginal tears, which would be treated by nurses, and a 12.7% incidence of grade II–III lacerations. There were no differences between the groups with or without active TENS. Tischendorf et al. [36] also suggested that the reduction of pain achieved through TENS could promote lacerations.

On the other hand, TENS represents an alternative method in pain relief for those women who wish to have a natural delivery and when epidural analgesia is not available or contraindicated. According to this idea, the review of Bedwell al. [9] argued that some women wish to have methods to enable them to cope with pain, which they see as an integral and necessary part of labour. Nevertheless, our results are not without their own limitations. We did not compare TENS with other non-pharmacological pain relief methods.

A further major weakness of our study was that we did not evaluate patient anxiety, in spite of its potential role as a confounding factor in studies on pain reduction interventions. Women during labour experience significant levels of anxiety with repercussions on pain perception and satisfaction. Psychological variables are also likely to play a role. Anxiety and depression have been considered from a physiological standpoint, but they are also likely to affect maternal behaviour during the birth. Maternal anxiety is associated with lower self-efficacy and confidence, a greater perceived threat, and increased pessimism [37–39]. Mothers who are in pain prenatally may be more anxious of the birth experience and enter childbirth with an increased level of physiological arousal as a consequence of both the pain they are experiencing and the psychological implications. Increased physiological arousal during labour has also been associated with reducing contractions and increasing the duration of labour and foetal distress, thus increasing the likelihood of an intervention. In the context of mothers who have prenatal pain may be more anxious about the experience of childbirth, for this reason we talk about offering a pre-labor experience with less pain. Since the TENS is an economical and easy-to-use device, its use could be taught to women for their use at home.

## Conclusions

TENS is a non-pharmacologic, effective, and safe option for pain relief during labour. With the use of high frequencies modified in time (80–100 Hz) as well as a high pulse width (350 μs), results showed a clinically and statistically significant difference. Pain relief during labour increased satisfaction levels in general, not only the satisfaction results connected with the moment of childbirth. Despite the results obtained, we must bear in mind that there are many factors that influence pain during childbirth. Therefore, it cannot be assumed that relief of pain and the level of satisfaction are due solely to the use of TENS.

## Abbreviations

CONSORT: Consolidated Standards of Reporting Trials
TENS: Transcutaneous electrical nerve stimulation
VAS: Visual analogue scale

## References

[1] Lowe NK (2006). The nature of labor pain. Am J Obstet Gynecol.

[2] Brown A, Johnston R (2013). Maternal experience of musculoskeletal pain during pregnancy and birth outcomes: significance of lower back and pelvic pain. Midwifery.

[3] Jones L, Othman M, Dowswell T, Alfirevic Z, Gates S, Newburn M, et al. Pain management for women in labour: an overview of systematic reviews. Cochrane Database Syst Rev. 2012;(3):CD009234. 10.1002/14651858.CD009234.pub2. 10.1002/14651858.CD009234.pub2 PMC7132546 22419342

[4] Melzack R, Wall PD (1965). Pain mechanisms: a new theory. Science.

[5] Simkin P, Bolding A (2004). Update on nonpharmacologic approaches to relieve labor pain and prevent suffering. J Midwifery Womens Health.

[6] Tournaire M, Theau-Yonneau A (2007). Complementary and alternative approaches to pain relief during labor. Evid Based Complement Alternat Med.

[7] Van der Spank JT, Cambier DC, De Paepe HM, Danneels LA, Witvrouw EE, Beerens L (2000). Pain relief in labour by transcutaneous electrical nerve stimulation (TENS). Arch Gynecol Obstet.

[8] Chao A, Chao A, Wang T, Chang YC, Peng HH, Chang SD (2007). Pain relief by applying transcutaneous electrical nerve stimulation (TENS) on acupuncture points during the first stage of labor: a randomized double-blind placebo-controlled trial. Pain.

[9] Bedwell C, Dowswell T, Neilson JP, Lavender T (2011). The use of transcutaneous electrical nerve stimulation (TENS) for pain relief in labour: a review of the evidence. Midwifery.

[10] Dowswell T, Bedwell C, Lavender T, Neilson JP. Transcutaneous electrical nerve stimulation (TENS) for pain management in labour. Cochrane Database Syst Rev. 2009;(2):CD007214. 10.1002/14651858.CD007214.pub2. 10.1002/14651858.CD007214.pub2 PMC4297467 19370680

[11] Wang SM, Kain ZN, White P (2008). Acupuncture analgesia: II. Clinical considerations. Anesth Analg.

[12] Carroll D, Tramer M, McQuay H, Nye B, Moore A (1997). Transcutaneous electrical nerve stimulation in labour pain: a systematic review. BJOG.

[13] Steer P, Chamberlain G, Wraight A, Steer P (1993). The methods of pain relief used. Pain and its relief in childbirth: the result of a National Survey conducted by the National Birthday Trust.

[14] Van der Ploeg JM, Vervest HA, Liem AL, Schagen van Leeuwen JH (1996). Transcutaneous nerve stimulation (TENS) during the first stage of labour: a randomized clinical trial. Pain.

[15] Van der Spank JT, Cambier DC, De Paepe HM, Danneels LA, Witvrouw EE, Beerens L (2000). Pain relief in labour by transcutaneous electrical nerve stimulation (TENS). Arch Gynecol Obstet.

[16] Haahr M. Random. org: True random number service. School of Computer Science and Statistics, Trinity College, Dublin, Ireland. 2010. Website (http://www.random.org). Accessed 10 Nov 2016.

[17] Bjordal JM, Johnson MI, Ljunggreen AE (2003). Transcutaneous electrical nerve stimulation (TENS) can reduce postoperative analgesic consumption. A meta-analysis with assessment of optimal treatment parameters for postoperative pain. Eur J Pain.

[18] Radhakrishnan R, Sluka KA (2005). Deep tissue afferents, but not cutaneous afferents mediate transcutaneous electrical nerve stimulation-induced antihyperalgesia. J Pain.

[19] Claydon LS, Chesterton LS, Barlas P, Sim J (2008). Effects of simultaneous dual-site TENS stimulation on experimental pain. Eur J Pain.

[20] Bernstein SL, Bijur PE, Gallagher EJ (2006). Relationship between intensity and relief in patients with acute severe pain. Am J Emerg Med.

[21] Gallagher EJ, Liebman M, Bijur PE (2001). Prospective validation of clinically important changes in pain severity measured on a visual analog scale. Ann Emerg Med.

[22] Todd KH, Funk KG, Funk JP, Bonacci R (1996). Clinical significance of reported changes in pain severity. Ann Emerg Med.

[23] Santana LS, Gallo RB, Ferreira CH, Duarte G, Quintana SM, Marcolin AC (2016). Transcutaneous electrical nerve stimulation (TENS) reduces pain and postpones the need for pharmacological analgesia during labour: a randomised trial. J Physiother.

[24] Janssen PA, Dennis CL, Reime B (2006). Development and psychometric testing of The Care in Obstetrics: Measure for Testing Satisfaction (COMFORTS) scale. Res Nurs Health.

[25] Montes MLV, Muñoz MS, del Rey GM, Ferrer RMR, Plaza CA, Santos IM (2012). Adaptación cultural y validación al español en la escala COMFORTS de satisfacción de las mujeres con los cuidados en el parto y puerperio. Metas de enfermería.

[26] Bundsen P, Ericson K, Peterson L, Thiringer K (1982). Pain relief in labour by transcutaneous electrical nerve stimulation: testing of a modified stimulation technique and evaluation of the neurological and biochemical condition of the newborn infant. Acta Obstet Gynecol Scand.

[27] Chamberlain G, Wraight A, Steer P (1993). The methods of pain relief used. Pain and its relief in childbirth: the result of a National Survey conducted by the National Birthday Trust.

[28] Saxena KN, Shokeen S, Taneja B (2016). Comparative evaluation of efficacy of transcutaneous electrical nerve stimulation administered by dermatomal stimulation versus acupuncture points stimulation. Northern J ISA.

[29] Bloodworth DM, Nguyen BN, Garver W, Moss F, Pedroza C, Tran T (2004). Comparison of stochastic vs. conventional transcutaneous electrical stimulation for pain modulation in patients with electromyographically documented radiculopathy. Am J Phys Med Rehabil.

[30] Tong KC, Lo SK, Cheing GL (2007). Alternating frequencies of transcutaneous electric nerve stimulation: does it produce greater analgesic effects on mechanical and thermal pain thresholds?. Arch Phys Med Rehabil.

[31] Chen C, Johnson MI (2009). An investigation into the effects of frequency-modulated transcutaneous electrical nerve stimulation (TENS) on experimentally-induced pressure pain in healthy human participants. J Pain.

[32] Johnson MI (2001). Transcutaneous electrical nerve stimulation (TENS) and TENS-like devices: do they provide pain relief?. Pain.

[33] Sluka KA, Walsh D (2003). Transcutaneous electrical nerve stimulation: basic science mechanisms and clinical effectiveness. J Pain.

[34] Kaplan B, Rabinerson D, Lurie S, Bar J, Krieser U, Neri A (1998). Transcutaneous electrical nerve stimulation (TENS) for adjuvant pain-relief during labour and delivery. Int J Gynaecol Obstet.

[35] Besnier F, Sénard JM, Grémeaux V, Riédel M, Garrigues D, Guiraud T (2017). The efficacy of transcutaneous electrical nerve stimulation on the improvement of walking distance in patients with peripheral arterial disease with intermittent claudication: study protocol for a randomised controlled trial: the TENS-PAD study. Trials.

[36] Tischendorf D (1986). Transcutaneous electrical nerve stimulation (TENS) in obstetrics. Zentralbl Gynakol.

[37] Williams K, Galliher R (2006). Predicting depression and self esteem from social connectedness, support and competence. J Soc Clin Psychol.

[38] Suls J, Martin R (2005). The daily life of the garden-variety neurotic: reactivity, stressor exposure, mood spill-over and maladaptive coping. J Pers.

[39] Ebstrup JF, Eplov LF, Pisinger C, Jørgensen T (2001). Association between the five factor personality traits and perceived stress: is the effect mediated by general self-efficacy?. Anxiety Stress Coping.

